# The Keystone State

**Published:** 1897-03

**Authors:** 


### THE KEYSTONE STATE.

PENNSYLVANIA'S State Board of Health will have many measures before the Legislature at this session. This body has many needs, all of which are of public import and necessity. In no great State like the Keystone has such niggardliness in appropriations been accorded as has been doled out to this body. Limited powers and a narrow, short-sighted policy have prevailed in recognition of the needs of this board, and much good that should have inured to the people of this State has been denied to our citizens. With an efficient board, an earnest set of representative men, and a hard-working, faithful, intelligent secretary this should no longer be, and the members of the veterinary profession, in their close relations with sanitary science and police measures, should interest themselves in these proposed enactments and urge upon their representatives a larger appropriation, greater power, and the removal of dangerous and needless restrictions, which contribute much to retard the work of this board in the progress of better sanitary regulations, which are the evidences of advancement to a higher civilization.

